# Genetic control of inflorescence architecture in legumes

**DOI:** 10.3389/fpls.2015.00543

**Published:** 2015-07-21

**Authors:** Reyes Benlloch, Ana Berbel, Latifeh Ali, Gholamreza Gohari, Teresa Millán, Francisco Madueño

**Affiliations:** ^1^Molecular Genetics Department, Center for Research in Agricultural Genomics, Consortium CSIC-IRTA-UAB-UB, Parc de Recerca Universitat Autònoma de BarcelonaBarcelona, Spain; ^2^Instituto de Biología Molecular y Celular de Plantas, Consejo Superior de Investigaciones Científicas – Universidad Politécnica de ValenciaValencia, Spain; ^3^Departamento de Genética, Universidad de CórdobaCórdoba, Spain

**Keywords:** legumes, pea, inflorescence architecture, meristem identity, *AP1*, *TFL1*, *VEG1*

## Abstract

The architecture of the inflorescence, the shoot system that bears the flowers, is a main component of the huge diversity of forms found in flowering plants. Inflorescence architecture has also a strong impact on the production of fruits and seeds, and on crop management, two highly relevant agronomical traits. Elucidating the genetic networks that control inflorescence development, and how they vary between different species, is essential to understanding the evolution of plant form and to being able to breed key architectural traits in crop species. Inflorescence architecture depends on the identity and activity of the meristems in the inflorescence apex, which determines when flowers are formed, how many are produced and their relative position in the inflorescence axis. *Arabidopsis thaliana*, where the genetic control of inflorescence development is best known, has a simple inflorescence, where the primary inflorescence meristem directly produces the flowers, which are thus borne in the main inflorescence axis. In contrast, legumes represent a more complex inflorescence type, the compound inflorescence, where flowers are not directly borne in the main inflorescence axis but, instead, they are formed by secondary or higher order inflorescence meristems. Studies in model legumes such as pea (*Pisum sativum*) or *Medicago truncatula* have led to a rather good knowledge of the genetic control of the development of the legume compound inflorescence. In addition, the increasing availability of genetic and genomic tools for legumes is allowing to rapidly extending this knowledge to other grain legume crops. This review aims to describe the current knowledge of the genetic network controlling inflorescence development in legumes. It also discusses how the combination of this knowledge with the use of emerging genomic tools and resources may allow rapid advances in the breeding of grain legume crops.

## Introduction: Inflorescence Architecture

One of the most interesting features of plant development is the fact that all aerial parts of the plant body are generated from the activity of the shoot apical meristem (SAM). The SAM is located at the tip of the plant shoot and contains a central pool of stem cells that are able to self-maintain together with peripheral dividing cells required for organ initiation (Steeves and Sussex, 1989). During the vegetative phase, the SAM generates leaf primordia with axillary vegetative shoots, in a sequential manner until floral transition is attained. Upon floral transition, the SAM becomes an inflorescence meristem that, either directly or in flower-bearing shoots, produces the floral meristems that form the flowers. The position where meristems are formed in the inflorescence apex and the activity of those meristems determines to a high degree the architecture of the inflorescence, the part of the plant that bears the flowers (Weberling, 1989a; Benlloch et al., 2007; Prusinkiewicz et al., 2007).

**FIGURE 1:**
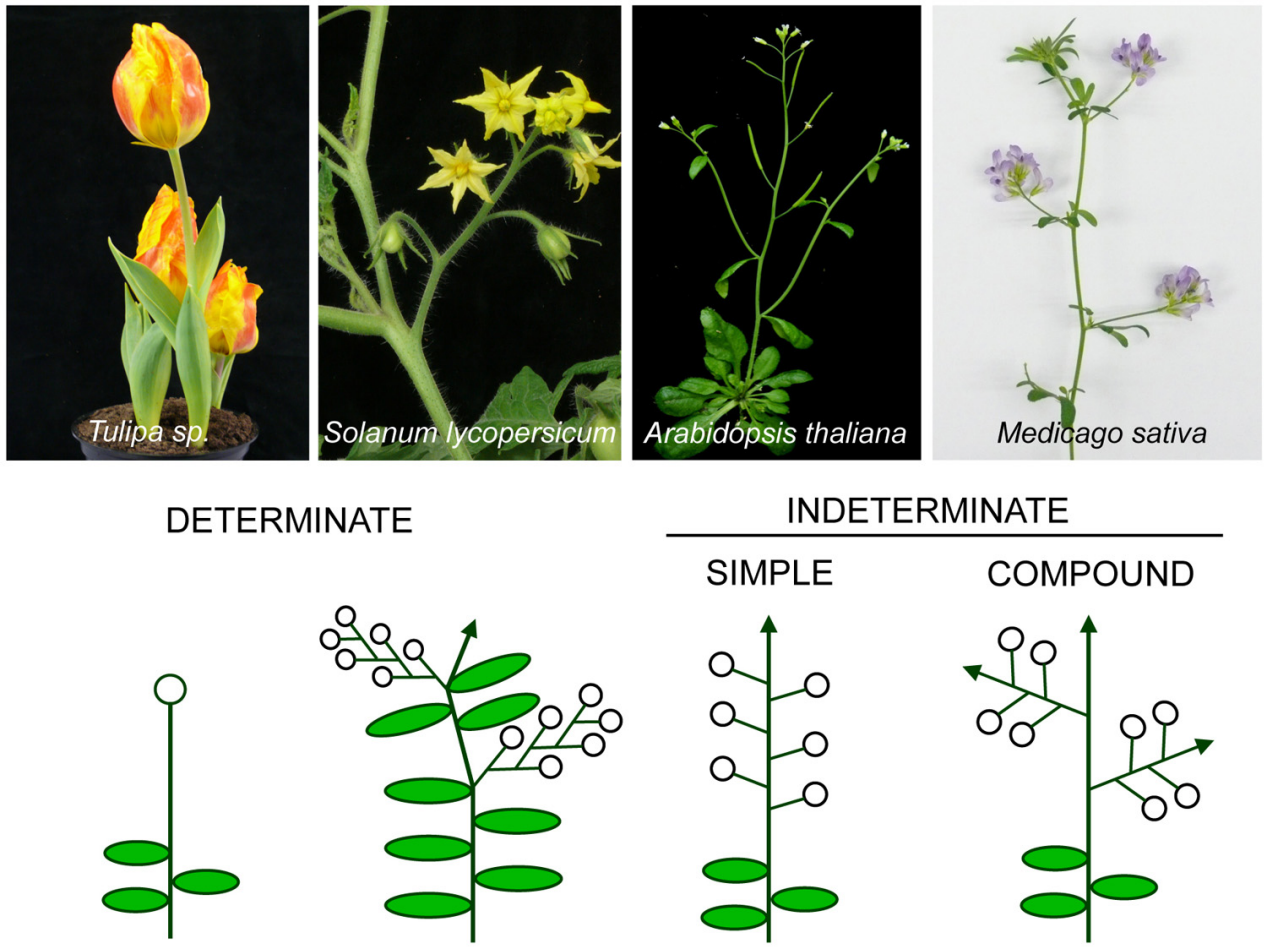
**Different types of inflorescence architecture.** Images of plant species representative of main inflorescence types **(top)** and the corresponding diagrams **(below)** of the architecture of their inflorescences. Open circles represent flowers and arrows represent indeterminate shoots.

A basic classification divides inflorescences into two groups, depending on whether the primary inflorescence axis terminates into a flower or not. According to this classification, *determinate* inflorescences are those where, after floral transition, the SAM acquires the identity of a floral meristem, which forms a terminal flower (TFL; Weberling, 1989a). This type of inflorescence includes extremely simple architectures, such as that of *Tulipa* sp, to more complex forms such as the cymes, found for instance, in some Solanaceae species (Lippman et al., 2008), where after formation of the TFL by the primary axis growth continues from lateral axes that repeat this pattern (**Figure 1**; Weberling, 1989a). On the contrary, in *indeterminate* inflorescences the SAM is never converted into a floral meristem and the inflorescence meristem continues producing floral meristems until senescence, as for example, occurs in the model plant species *Arabidopsis thaliana* (**Figure 1**; Weberling, 1989a; Benlloch et al., 2007).

According to another main classification, *Arabidopsis* is also an example of a *simple* inflorescence, as its flowers are directly formed in the primary inflorescence axis. In contrast, other plants have evolved to a more complex architecture and have *compound* inflorescences (**Figure 1**; Weberling, 1989a). In compound inflorescences, the flowers are not formed in the primary inflorescence axes but, instead, they are formed in secondary or higher order axes (**Figure 1**). Compound inflorescences are typical, for instance, of grasses and legumes (Weberling, 1989b; Kellogg, 2007).

The architecture of the inflorescence, which conditions how many flowers (and therefore, fruits and seeds) are produced, and their position in the plant, has a profound impact on key agronomical aspects such as crop management, yield and yield stability. For instance, in crops such as tomato and grain legumes, determinate varieties have been traditionally selected because they show favorable traits for an efficient cultivation and harvest. Determinate varieties often display a shorter flowering time and earlier maturation and they are usually more compact, facilitating large-scale harvesting (Tian et al., 2010; Park et al., 2014). Taken into account the great economic importance of grain legumes, which include broadly used species for food and feed, it is of great interest to understand the genetic bases of inflorescence architecture in these species. In this context, genes controlling inflorescence development are instrumental for the generation of breeding and biotechnological tools to design new legume crops better adapted to different environmental conditions.

In this review, we describe the current knowledge on the genetic control of inflorescence architecture in grain legumes, and discuss the biotechnological potential of this knowledge for the development and selection of more productive and sustainable legume crop varieties.

## Genetic Network Controlling Meristem Identity in the *Arabidopsis* Inflorescence

A main factor that shapes inflorescence architecture is the identity of the meristems produced in the inflorescence apex, which determines the relative position where flowers are formed.

**FIGURE 2:**
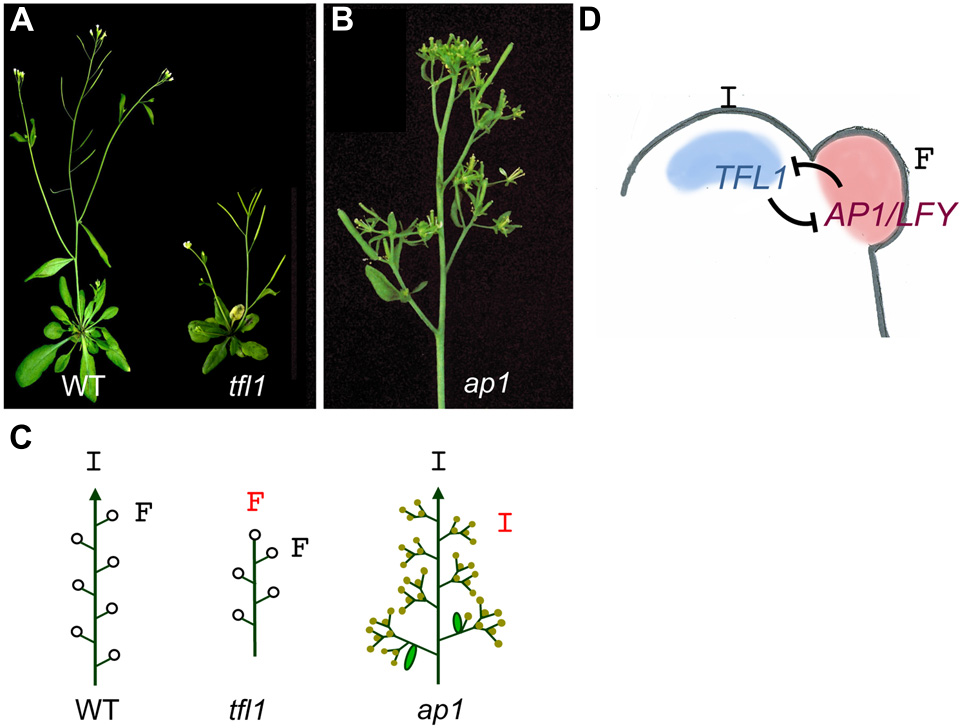
**Meristem identity genes in *Arabidopsis*. (A)** Images of wild-type (WT) and tfl1 mutant plants. While in the WT the main inflorescence and the lateral inflorescences (appearing in the axil of cauline leaves) show indeterminate growth, in the *tfl1* mutant the main inflorescence ends into a terminal flower (a fruit in this image) and lateral branches are replaced by solitary flowers. **(B)** Inflorescence of an *ap1* mutant. Individual flowers are replaced by branched structures. **(C)** Diagrams of meristem identity in the inflorescences of the wild-type and the *tfl1* and *ap1* mutants. In *tfl1*, the indeterminate inflorescence apex (I) is replaced by a terminal flower (F) while in *ap1*, the flowers are replaced by inflorescence-like structures. Arrowheads, indeterminate shoot; open circles, flowers, closed circles, abnormal flowers. **(D)** Model for specification of meristem identity in the simple inflorescence of *Arabidopsis*. In the *Arabidopsis* inflorescence apex, *TFL1* expression in the inflorescence meristem (I) and *AP1* and *LFY* expression in the floral meristem (F) are required for these meristems to acquire their identity. Expression of these genes in their correct domains is maintained by mutual repressive interactions.

*Arabidopsis thaliana* is one of the best-known examples of simple indeterminate inflorescences. In *Arabidopsis*, upon floral transition, the vegetative meristem becomes an inflorescence meristem, which produces floral meristems laterally (**Figures 1** and **2**). The development of the *Arabidopsis* inflorescence can be mostly explained by the function and mutual regulation of three genes: *TERMINAL FLOWER 1* (*TFL1*), *LEAFY* (*LFY*), and *APETALA 1* (*AP1*) (Shannon and Meeks-Wagner, 1993; Liljegren et al., 1999; Blazquez et al., 2006). These three genes act as opposing forces maintaining the balance between inflorescence and floral meristem identity at the inflorescence apex (Ratcliffe et al., 1999; Blazquez et al., 2006).

### LEAFY

The *LFY* and *AP1* genes are essential for the specification of floral meristem identity in *Arabidopsis*. *LFY* codes for a plant-specific transcription factor that is expressed at very early stages in the flanks of the inflorescence meristem, at the floral “anlagen” (the groups of cells that will form the floral meristems) directing these incipient primordia to a floral meristem fate (Schultz and Haughn, 1991; Huala and Sussex, 1992; Weigel et al., 1992; Maizel et al., 2005). Hence, meristems produced by the SAM after floral transition in loss-of-function *lfy* mutants have problems to acquire floral identity and retain features typical of inflorescence meristems. This results in replacement of the first flowers on the inflorescence stem by shoots. Nevertheless, at later stages of development, the inflorescencs of *lfy* mutants produce flower-like structures, which show that, in addition to *LFY*, other genes participate in the specification of floral meristem identity.

### APETALA1

The *AP1* gene codes for a MADS-box transcription factor also required for floral meristem identity specification (Mandel et al., 1992; Weigel and Meyerowitz, 1993). *AP1* transcription is directly activated by LFY at stage 1 floral meristems (Wagner et al., 1999). In *ap1* mutants the first flowers on the inflorescence stem are replaced by shoots and their flowers display severe morphological and homeotic alterations. The sepals of the *ap1* mutant flowers are replaced by bract-like organs and, in the axils of these organs, secondary flowers are produced, which again may produce axillary flowers (**Figure 2**; Irish and Sussex, 1990; Bowman et al., 1993). The formation of bract-like organs and ramified flowers indicates a partial reversion from floral fate to inflorescence. This incomplete reversion suggests that other genes may act redundantly with *AP1* in the specification of floral meristem fate. In *Arabidopsis*, this redundant function is played by the *CAULIFLOWER* (*CAL*) gene, a paralogue of *AP1* that is also expressed in early floral meristems as a result of LFY direct activation (Bowman et al., 1993; Mandel and Yanofsky, 1995; William et al., 2004). The combination of *ap1* and *cal* mutations results in a complete absence of floral meristem identity acquisition. Thus, in double *ap1 cal* mutants, inflorescence meristems produce new meristems that completely fail to acquire floral fate behaving like new inflorescence meristems that continue to divide, producing proliferating structures with cauliflower morphology (Bowman et al., 1993; Kempin et al., 1995; Mandel and Yanofsky, 1995).

### TERMINAL FLOWER 1

The *TFL1* gene encodes for a phosphatidyl-ethanolamine-binding protein (PEBP) and it is expressed in a subset of cells of the SAM at low level during vegetative stage (**Figure 2**; Bradley et al., 1997; Ohshima et al., 1997). *TFL1* expression increases after floral transition and TFL1 protein acts as a signal controlling inflorescence meristem identity. Mutations in the *TFL1* gene cause a conversion of the inflorescence meristems into floral meristems, producing the abrupt termination of the main inflorescence stem in a TFL and the substitution of lateral branches by solitary axillary flowers (**Figure 2**; Shannon and Meeks-Wagner, 1991; Alvarez et al., 1992; Schultz and Haughn, 1993). Therefore, the *tfl1* mutation changes the *Arabidopsis* inflorescence from an indeterminate to a determinate type. In agreement with its expression in the vegetative meristem, TFL1 also has a role controlling the length of the vegetative phase, acting as a repressor of flowering. Thus, *tfl1* mutant plants flower earlier that the wild type, with a reduction in the number of leaves and branches produced in the main stem (Shannon and Meeks-Wagner, 1991; Schultz and Haughn, 1993).

Analyses of genetic interactions between these mutants, together with the expression patterns of *TFL1*, *LFY*, and *AP1*, led to a model for the control of meristem identity in the inflorescence of *Arabidopsis* (**Figure 2**; Liljegren et al., 1999; Ratcliffe et al., 1999; Blazquez et al., 2006). According to this model, inflorescence meristem identity in *Arabidopsis* is maintained by the activity of TFL1, which represses *AP1* and *LFY* genes in the inflorescence meristem, preventing early inflorescence termination. In fact, in *tfl1* mutants, *AP1* and *LFY* are ectopically expressed in the inflorescence meristems, which in turn acquire floral fate and produce terminal and axillary flowers (Mandel et al., 1992; Weigel et al., 1992; Bradley et al., 1997). Conversely, *LFY* and *AP1* are expressed in the meristems produced at the flanks of the inflorescence meristem, which thus acquire floral identity and form the flowers. LFY and AP1 repress *TFL1* in the newly formed floral meristems, allowing up-regulation of floral organ identity genes and hence the formation of flowers (Parcy et al., 1998; Liljegren et al., 1999; Wagner et al., 1999; Kaufmann et al., 2010). This simple model of mutual repression between TFL1 and LFY/AP1 elegantly explains the maintenance of the indeterminate inflorescence meristem in *Arabidopsis* and the formation of floral meristems at its flanks.

## Genetic Network Controlling Meristem Identity in the Legume Inflorescence

**FIGURE 3:**
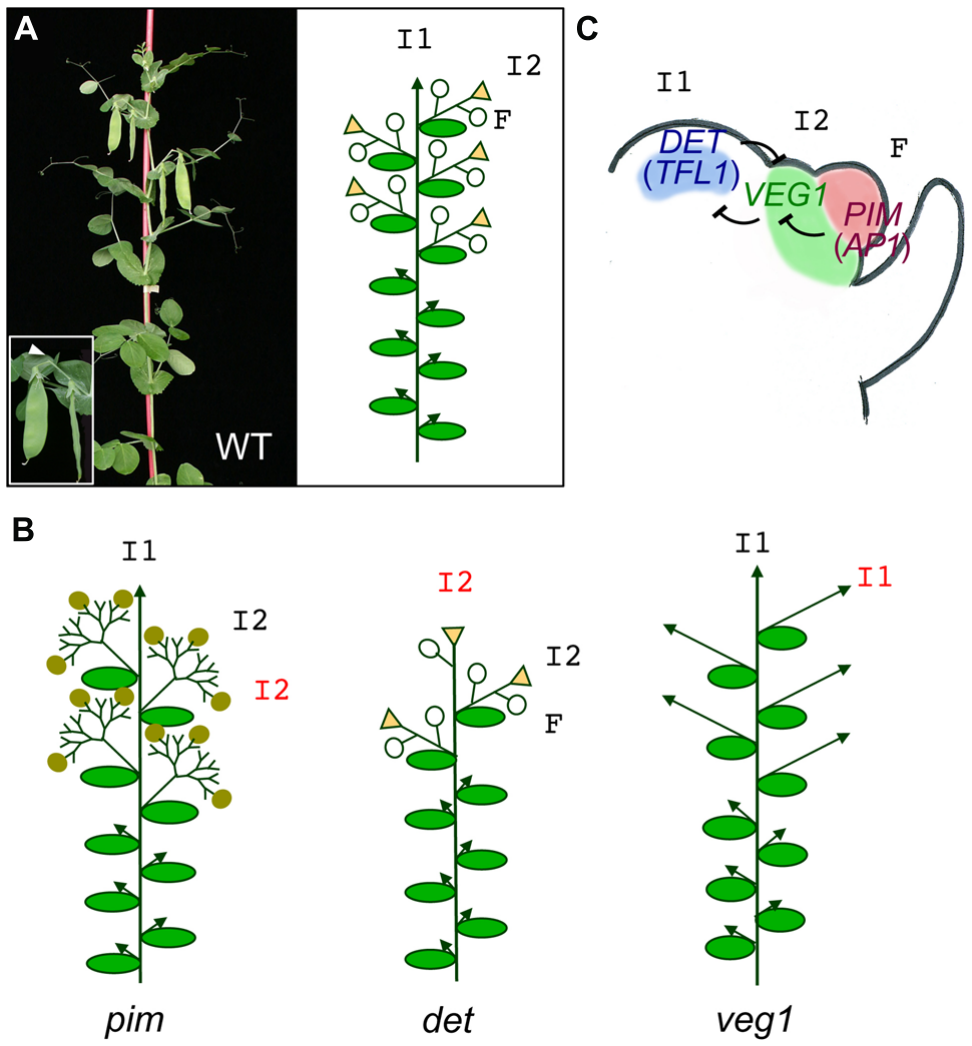
**Meristem identity genes in pea. (A)** Picture and diagram of a pea WT plant. The main primary inflorescence (I1) shows indeterminate growth (arrowhead). Upper nodes of the plant contain secondary inflorescences (I2) which produce 1–2 flowers (F, open circles) and terminate into a stub (triangles). The inset shows a close up of a secondary inflorescence with two flowers (pods) and the stub (arrowhead). **(B)** Diagrams of meristem identity of the *pim*, *det*, and *veg1* mutants. In the *pim* mutant, flowers are replaced by proliferating I2s with abnormal flowers (closed circles). In the *det* mutant, the primary inflorescence is replaced by a terminal secondary inflorescence. In the *veg1* mutant, the I2s are replaced by vegetative branches with I1 identity. **(C)** Model for specification of meristem identity in the compound pea inflorescence. In the pea inflorescence apex, *DET* expression in the primary inflorescence meristem (I2), *VEG1* in the secondary inflorescence meristem (I2) and *PIM* in the floral meristem (F) are required for these meristems to acquire their identity. Expression of these genes in their correct domains is maintained by a network of mutual repressive interactions.

As mentioned above, legumes are characterized by a compound indeterminate inflorescence (Weberling, 1989b; Benlloch et al., 2007; Prenner, 2013; Hofer and Noel Ellis, 2014). The ontogeny of the compound inflorescence has been described in detail in pea (*Pisum sativum*; Singer et al., 1999). Briefly, the SAM undergoes a transition from a vegetative meristem to a primary inflorescence (I1) meristem, with indeterminate growth. This I1 meristem, instead of producing floral meristems at its flanks, as in the case of *Arabidopsis*, produces secondary inflorescence meristems (I2), which in turn will generate floral meristems (F). In pea, the I2 usually produces 1-2 floral meristems before it ceases growing, forming a residual organ or stub (**Figure 3**). Therefore, the appearance of the I2 meristem supposes an additional level of complexity in the legume inflorescence, as compared to *Arabidopsis*, and different genes have been coopted to orchestrate the development of the compound inflorescence in legumes.

Among legumes, pea is the species where genetics of inflorescence development is best understood. In this section, we will review the current knowledge on the genes controlling floral meristem identity, as well as primary and secondary inflorescence meristem identity, primarily in this species and then extending to what is known for other legume species.

### Floral Meristem Identity

In pea, floral meristem identity is controlled by the homologs of the *LFY* and *AP1* genes from *Arabidopsis*. The *PROLIFERATING INFLORESCENCE MERISTEM* (*PIM/PEAM4*) and *UNIFOLIATA* (*UNI*) genes have been characterized as homologs to *AP1* and *LFY*, respectively (Hofer et al., 1997; Berbel et al., 2001; Taylor et al., 2002). The function of *PIM* and *UNI* in the control of inflorescence architecture is similar to their counterparts in *Arabidopsis*, although some functional differences are also found, possibly accounting for the more complex inflorescence and flower development exhibited by legumes.

#### PROLIFERATING INFLORESCENCE MERISTEM

The pea MADS-box gene *PIM*/*PEAM4* is specifically expressed in floral meristems (Berbel et al., 2001, 2012; Taylor et al., 2002). Its pattern of expression is similar to *AP1*, being uniformly expressed in floral meristems at early stages and restricted to the sepal and petal primordia in later stages. Overexpression of *PIM* in *Arabidopsis* causes early flowering and, often, the formation of a TFL and replacement of branches by axillary flowers. These phenotypic alterations are also observed in 35S::AP1 plants and indicate that *PIM* specifies floral meristem identity, being its expression in the inflorescence meristem sufficient to convert it into a floral meristem. *pim* mutants do not show alterations in vegetative traits and I1 and I2 meristems are correctly specified. However, *pim* I2 meristems, rather than producing floral meristems, produce new I2 meristems in a reiterative manner (**Figure 3**; Taylor et al., 2002; Berbel et al., 2012), somehow resembling the proliferative inflorescences of the *Arabidopsis ap1 cal* double mutant (Kempin et al., 1995). Likewise, the I2 meristems of the *pim* mutants are eventually able to produce floral meristems; in these cases the meristems only acquire partial floral fate, as indicated by the production of flowers with bract-like organs and other floral identity defects (Taylor et al., 2002).

The function of *PIM* seems to be conserved in other grain legumes. *PIM* homologs have also been described in *Lotus japonicus*, *LjAP1a* and *LjAP1b*, and in *Medicago truncatula*, *MtPIM* (Dong et al., 2005; Benlloch et al., 2006). These homologs show an expression pattern during floral meristem initiation and development very similar to that of *PIM*. The *Medicago mtpim* mutant also exhibits a proliferating inflorescence phenotype, somehow more severe than the pea mutants, where floral meristems are replaced by proliferating I2 meristems (Benlloch et al., 2006).

#### UNIFOLIATA

Another gene with a key function in the in the initiation of floral meristems in pea is the *LFY* homolog *UNI* (Hofer et al., 1997). In the loss-of-function *uni* mutants, floral meristems are not correctly specified and rather than flowers they produce proliferating structures, mainly formed by sepals and carpels. These structures derive from flowers with severe loss of determinacy, where supernumerary flowers reiteratively arise in the axil of sepals, apparently replacing petals and stamens (Hofer et al., 1997). This phenotype partly resembles that of *lfy* mutants in *Arabidopsis*, whose flowers never form petals or stamens. Nevertheless, the *uni* phenotype is less severe than that of *lfy* mutants as, in the strict sense, replacement of flowers by branches is not observed in *uni* mutants; instead, the proliferating *uni* flowers rather resemble the branched flowers of *Arabidopsis ap1* mutants. Mutants in the *UNI* homologs of *L. japonicus*, *LjLFY* and *M. truncatula*, *SINGLE LEAFLET1* (*SGL1*) have also been described and both produce flowers with a very similar phenotype to those of *uni* (Dong et al., 2005; Wang et al., 2008a).

Expression of *UNI* in pea floral meristems is detected in developing floral organ primordia, declining as they expand. In *L. japonicus* and *M. truncatula* the *UNI* homologs also show expression in young floral organ primordia. In addition, although *uni* mutants do not show apparent defects in I2 meristem specification, expression of *UNI* genes was also described in I2 meristems in pea and *M. truncatula*.

*UNI* has an additional function in the control of the compound leaf development in pea, as shown by the phenotype of *uni* mutants, where the complexity of the leaves is strongly reduced (Hofer et al., 1997; Gourlay et al., 2000). The number of leaflets is reduced in *uni* mutant and tendrils are not formed. This function seems conserved in other grain legumes from the IRCL clade, as mutants in the *L. japonicus* and *M. truncatula UNI* homologs, *LjLFY* and *SLG1*, also show a strong reduction in the complexity of their leaves (Dong et al., 2005; Wang et al., 2008a). In this context, compound leaves are interpreted as “partially indeterminate” and have been proposed that *UNI* would have a role in the control of determinacy not only of floral meristems but also of leaf primordia (Hofer and Noel Ellis, 2014).

### I1 Meristem Identity

#### DETERMINATE

As described above, grain legumes have an indeterminate inflorescence, where the I1 meristem does not form a TFL but continues producing lateral I2s until it ceases growing (**Figures 1** and **3**). Pea mutants in the *DETERMINATE* (*DET*) gene have a determinate inflorescence that produces 1-2 normal lateral I2s and an apparent TFL, resembling the *Arabidopsis tfl1* mutant (Singer et al., 1990). However, a closer analysis reveals that the I1 meristem of *det* mutants do not directly form a (terminal) flower but, instead, it develops as a stem that produces a flower in a lateral position and terminates into a stub, like the I2s. This shows that in pea *det* mutants, rather than the conversion of the inflorescence meristem into floral meristem observed in *Arabidopsis tfl1* mutants, what really takes place is the conversion of the I1 meristem into an I2 meristem (**Figure 3**; Singer et al., 1990). The molecular identification of the *DET* gene showed that indeed it corresponds to a homolog of the *Arabidopsis TFL1* gene, which was named *PsTFL1a* (Foucher et al., 2003). *DET*/*PsTFL1a* is expressed only after floral transition, in the I1 meristem, in agreement with its function as an I1 meristem identity gene (Foucher et al., 2003; Berbel et al., 2012).

Mutants with determinate inflorescences have been described in other grain legumes. In the last years, the underlying mutations of some of these phenotypes have been identified, and these have been shown to affect *DET*/*PsTFL1a* homologs (Avila et al., 2007; Liu et al., 2010; Repinski et al., 2012; Dhanasekar and Reddy, 2014; Mir et al., 2014). An exception to this is the soybean *dt2* mutants, which show a semideterminate phenotype that is not caused by a mutation in a *TFL1*-like gene. The function of the *Dt2* gene will be discussed in next sections.

Another difference between the pea *det* mutant and the *Arabidopsis tfl1* mutant is that *tfl1* mutations, in addition to determination of the inflorescence, cause early flowering in *Arabidopsis*. Foucher et al. (2003) also showed that the early flowering phenotype of recessive mutations in the pea *LATE FLOWERING* loci (*LF*, described by Weller and Ortega, in this Research Topic) was due to mutations in another *TFL1-like* gene, *PsTFL1c*, a paralogue of *DET/PsTFL1a*. Interestingly, the pea *det lf* double mutant plants are early flowering and determinate, which strongly resembles the phenotype of *Arabidopsis tfl1* mutants. This has lead to the attractive idea that the *TFL1* function, which in *Arabidopsis* controls both the vegetative and the inflorescence phases (Ratcliffe et al., 1998), in pea would be divided between two genes, *DET* and *LF* (Foucher et al., 2003).

### I2 Meristem Identity

Particularly interesting is the specification of the secondary inflorescence (I2) meristem, as the formation of these meristems is crucial for the development of higher order inflorescences and hence for the formation of the characteristic legume compound inflorescences. Secondary inflorescence (I2) meristems do not form in simple inflorescences, indicating that new genetic functions must have appeared in evolution to direct the acquisition of I2 meristem identity. The identification of these novel genetic functions, and the characterization of their conservation across legumes, is important for a better understanding of compound inflorescence development.

#### VEGETATIVE1

The genetic basis of I2 meristem identity acquisition was elucidated by the analysis of a pea mutant in the *VEGETATIVE1* (*VEG1*) locus. *veg1* mutant plants present a extreme non-flowering phenotype: no flowers or floral organs are produced in *veg1* plants under any growing condition (Gottschalk, 1979; Reid and Murfet, 1984). Characterization of primary inflorescence markers in *veg1* mutant discarded the possibility that the floral transition was blocked or delayed in this mutant. Instead, the non-flowering phenotype of *veg1* is explained by a blockage on I2 meristem identity acquisition. Transition from vegetative to I1 meristem apparently takes place but the I1 meristem produces lateral meristems that, unable to acquire I2 identity, continue to develop as I1s, producing vegetative branches that replace I2 inflorescences (**Figure 3**; Gottschalk, 1979; Reid and Murfet, 1984; Berbel et al., 2012). In agreement with this, *DET* expression, which as discussed above specifies I1 meristem identity, was found in the lateral meristems produced at the flanks of the apical I1 meristem of the *veg1* plants, indicating that in wild-type (WT) pea, *VEG1* is required to confer I2 identity to these lateral meristems and that to achieve that, *VEG1* directly or indirectly represses *DET* expression in these meristems (Berbel et al., 2012).

*VEG1* was shown to correspond to *PsFULc*, a MADS-box gene belonging to the AGL79 clade of the *AP1*/*SQUA*/*FUL* genes (Berbel et al., 2012). In agreement with its proposed function in the control of I2 meristem identity, *VEG1/PsFULc* gene is expressed after floral transition in the inflorescence apex, specifically in I2 meristems, just before *PIM* upregulation and floral meristem development, and its expression is not detected in I1 or in floral meristems.

#### *VEGETATIVE2* and *GIGAS*

Two other genes are considered to participate in the control of I2 meristem identity in pea: *GIGAS* and *VEGETATIVE2* (Murfet and Reid, 1993; Beveridge and Murfet, 1996; Reid et al., 1996).

Plants with severe mutations in the *GIGAS* locus show an extreme non-flowering phenotype under long-day (LD) conditions. Similar to *veg1*, *gigas* mutants show apparently normal vegetative development, and later in development, the induction of inflorescence markers, such as upregulation of *DET* and bud outgrowth (Beveridge and Murfet, 1996; Hecht et al., 2011), indicating that transition from vegetative to I1 meristem also takes place in *gigas* mutants. However, expression of *PIM* and *VEG1* is never induced under LD in the inflorescence of the *gigas* mutants, which indicates that I2 specification does not take place (Hecht et al., 2011; Berbel et al., 2012). *GIGAS* corresponds to *PsFTa1*, one of the pea homologs of the *FLOWERING LOCUS T* (*FT*) gene in *Arabidopsis* (Hecht et al., 2011). FT has been identified as a major component of the florigen, the floral promoting signal that travels from the leaf to the apex and initiates the floral transition (reviewed in Pin and Nilsson, 2012). FT, in a complex with the bZIP transcription factor FD, directly upregulates the expression of floral genes, such as *AP1* (Abe et al., 2005; Wigge et al., 2005; Pin and Nilsson, 2012). However, in pea, and in most legume species, there are several *FT* genes, comprising three distinct clades; analysis of pea *FT* genes has revealed a much more complex regulation of photoperiodic flowering in pea compared to *Arabidopsis*, with different *FT* homologs expressed in leaf and/or apex, and displaying different responsiveness to photoperiod (described by Weller and Ortega, 2015, in this Research Topic).

Loss-of-function mutations in *VEG2* also cause a phenotype related to I2 meristem development. Thus, the *veg2-1* mutant displays a non-flowering phenotype similar to *veg1*, while *veg2-2*, a weaker allele, shows a delay in flowering and a conversion of I2 inflorescences into flower-bearing branch-like structures with indeterminate growth, which resemble the primary I1 inflorescence of WT plants (Murfet and Reid, 1993). *VEG2* has been recently shown to correspond to *PsFDa*, a pea orthologue of *FD* (Sussmilch et al., 2015). As in *gigas* mutants, expression of *PIM* and *VEG1* is never detected in the “inflorescence” apex of *veg2-1* mutant. As the VEG2/FDa protein is able to interact with GIGAS/FTa1, a likely possibility is that GIGAS and VEG2 form a transcriptional complex responsible for the upregulation of *VEG1*, which would not take place in *gi* or *veg2* mutants, thus explaining the absence of I2 development observed in these mutants.

### Model for Inflorescence Meristem Identity in Legumes

A genetic model for the specification of meristem identity in the pea compound inflorescences has been proposed based on the genetic studies described above and the existing knowledge on *Arabidopsis* inflorescence development (**Figure 3**).

In this model, primary and secondary inflorescence meristem identity is regulated by *DET* (*TFL1* homolog) and *VEG1*, respectively. *DET* and *VEG1* repress each other expression, ensuring the balance between the indeterminate development of the apical primary inflorescence (I1) and the formation of secondary inflorescence (I2) meristems at its flanks. The identity of the floral meristems produced by the I2 is controlled by *PIM*, the homolog of *AP1*. *PIM* expression in the newly formed floral meristems represses *VEG1* in this tissue, allowing floral development to proceed.

Consistent with this model, the missexpression of *VEG1* in the inflorescence apex of *det* mutant plants would cause the apical meristem to acquire I2 identity and to develop as a terminal I2. Conversely, in a *veg1* mutant, *DET* is ectopically expressed in the lateral meristems produced by the I1 and these lateral meristems then fail to acquire I2 identity, developing as I1 inflorescences. Finally, as it was also observed that loss-of-function of *PIM* leads to a missexpression of *VEG1* in the newly formed “floral” meristems, this ectopic VEG1 expression would explain why these meristems do not acquire floral fate but instead develop as I2 meristems. This simple model based on the DET/VEG1/PIM regulatory module (**Figure 3**) is a more complex version of the TFL1/LFY/AP1 model (**Figure 2**) that explains the development of the *Arabidopsis* simple inflorescence. With the introduction of a novel genetic function, represented by VEG1, which is placed in between the mutual antagonistic activities of DET and PIM, the pea model explains elegantly how the transient I2 meristem appears and, hence, the development of the compound inflorescence in legumes.

Though most grain legumes have compound inflorescences with a similar architecture to pea (Weberling, 1989b; Benlloch et al., 2007; Prenner, 2013; Hofer and Noel Ellis, 2014), the conservation of the DET/VEG1/PIM regulatory network among the legume family remains to be proved. Diverse evidence suggests that, indeed, the properties and architecture of this network could be conserved among grain legumes. The determinate growth habit caused by mutations in *DET/TFL1*-homologs in other grain legumes indicates that *DET* function is conserved in these species (Liu et al., 2010; Tian et al., 2010; Kwak et al., 2012; Repinski et al., 2012; Dhanasekar and Reddy, 2014). Also, the characterization of *MtPIM*, the *M. truncatula* homolog of *PIM*, indicates conservation of this function in *Medicago* (Benlloch et al., 2006). To our knowledge, there are no reported examples of *VEG1* loss-of-function mutants outside pea. Nevertheless, the dominant semideterminate inflorescence phenotype in the *dt2* soybean mutants has been recently associated to the overexpression of a *FULc/VEG1* homolog in the inflorescence apex of the mutant (Ping et al., 2014). According to the proposed model, elevated expression of a *VEG1* gene in the apical I1 meristem should repress *DET* expression and cause determination, hence the phenotype of the *dt2* mutants is consistent with the proposed repression of *DET* by VEG1 also being conserved in other grain legumes.

## Genetic Control of the Activity of Meristems in the Legume Inflorescence

Apart from meristem identity, which determines the relative position where flowers are formed in the inflorescence apex, a second factor with a key influence on the architecture of the inflorescence is the activity of the meristems. In plants with an indeterminate growth habit, such as grain legumes, the SAMs produce lateral structures, branches and flowers, while they remain active. Therefore, the number of secondary inflorescences (I2) produced by the primary inflorescence (I1), and the number of flowers produced by the secondary inflorescences, depends on for how long the I1 and I2 meristems, respectively, remain active.

The number of flowers produced in each I2, as well as the number of I2s produced by the primary inflorescence, is characteristic of each species or cultivar (Murfet, 1985; Annicchiarico and Iannucci, 2008; Smýkal et al., 2014). Thus, for instance, while pea usually produce between 1 and 2 flowers per secondary inflorescence, the secondary inflorescences of *Mellilotus officinalis* (yellow sweet clover) usually have 20–30 flowers.

### Number of Flowers per Secondary Inflorescence, Activity of the I2 Meristem

In spite of its possible influence on seed yield of crops, there are relatively few studies about the genetic control of flower number per I2 in legumes, which depends on the activity of the I2 meristem.

In the case of pea, where I2 meristems usually produce 1–2 flowers, several studies have identified loci responsible for limiting the number of floral meristems initiated by the I2 meristem before stub formation. Classical studies indicate that this trait is controlled by two genes, *Fn* and *Fna*, whose single recessive mutations cause an increase in the number of flowers per I2, being higher in the double recessive genotypes, the so-called multipod phenotype (White, 1917; Lamprecht, 1947). More recent studies describe that mutations in *NEPTUNE* (*NEP*), a gene represented by two recessive alleles, also causes a multipod phenotype (Singer et al., 1999), although the possible allelic relationship between *NEP* and the *Fn* and *Fna* genes has not been reported. In addition to these genetic factors, the number of flowers per I2 is also affected by growing conditions (Hole and Hardwick, 1976; Murfet, 1985; Singer et al., 1999) and mutations in the flowering time genes *HIGH RESPONSE* (*HR*) and *STERILE NODES* (*SN*), involved in photoperiod response, also strongly influence this trait, with the number of flowers being decreased by recessive *sn* alleles and increased by dominant *HR* alleles (Murfet, 1985; Reid et al., 1996; Weller et al., 2012; Liew et al., 2014).

Chickpea (*Cicer arietinum*) is the grain legume where genetics of number of flowers per I2 is possibly better understood. Most chickpea genotypes have only one flower per I2, and it has been proposed that this solitary flower could be a reduction of a multi-flowered ancestor (Prenner, 2013). Natural mutations that produce double triple and multi-flowers per I2 have been reported (Knights, 1987; Singh and Chaturvedi, 1998; Gaur and Gour, 2002). Two loci have been identified that control this trait: *Sfl* and *Cym*. Plants with a *Sfl*-*Cym* genotype are single-flowered, while the recessive *sfl^d^* and *sfl^t^* alleles cause a double-flower and triple-flower phenotype, respectively, the *sfl^d^* allele being dominant over *sfl^t^* (Srinivasan et al., 2006). The multi-flower phenotype is found in plants homozygous for the recessive allele in the *Cym* gene, which produces apparently cymose secondary inflorescences (Gaur and Gour, 2002).

As in the case of pea, number of flowers per I2 in chickpea is also affected by environmental conditions. Thus, *sfl^d^* allele showed higher penetrance and expressivity under soil moisture stress conditions (Sheldrake et al., 1978). Interestingly, more flowers do not directly mean more pods, and triple-flower plants can only develop two pods per I2, because one of the three flowers, with a different morphology, does not set pod (Srinivasan et al., 2006). In the same way, multi-flower plants can produce up to nine flowers per node but do not form more than four or five pods per I2 (Gaur and Gour, 2002; Srinivasan et al., 2006).

### Number of I2 Nodes in the Primary Inflorescence, Activity of the I1 Meristem

The flowering time genes *SN* and *HR* not only control the activity of the I2 meristem in pea, but also affect the duration of I1 meristem activity, since the number of I2 nodes produced before I1 meristem arrest is decreased by recessive *sn* alleles and increased by dominant *HR* alleles (Reid et al., 1996). Interestingly, some studies suggested that duration of I1 meristem activity could be uncoupled from flowering time (Reid, 1980).

In some cases, the number of I2 (flowering) nodes has been found to limit yield in legume crops (Roche et al., 1998; Kahlon et al., 2011), which indicates that the number of flowering nodes is a trait with the potential to improve yield. However, genes that specifically control the number of I2 nodes produced by the I1 meristem have not been identified so far.

## Inflorescence Traits Amenable to Improvement in Legume Crops

Inflorescence traits amenable to improvement in legumes could be divided into two categories: (1) traits related to the identity of the meristems in the inflorescence apex, and (2) traits related to the activity of the inflorescence meristems. In this section, we discuss genetic and/or biotechnological strategies to modify inflorescence traits that might be applicable in breeding programs either to synchronize maturity facilitating mechanical harvesting or to improve and stabilize yields.

### Traits Related to Inflorescence Meristem Identity

**FIGURE 4:**
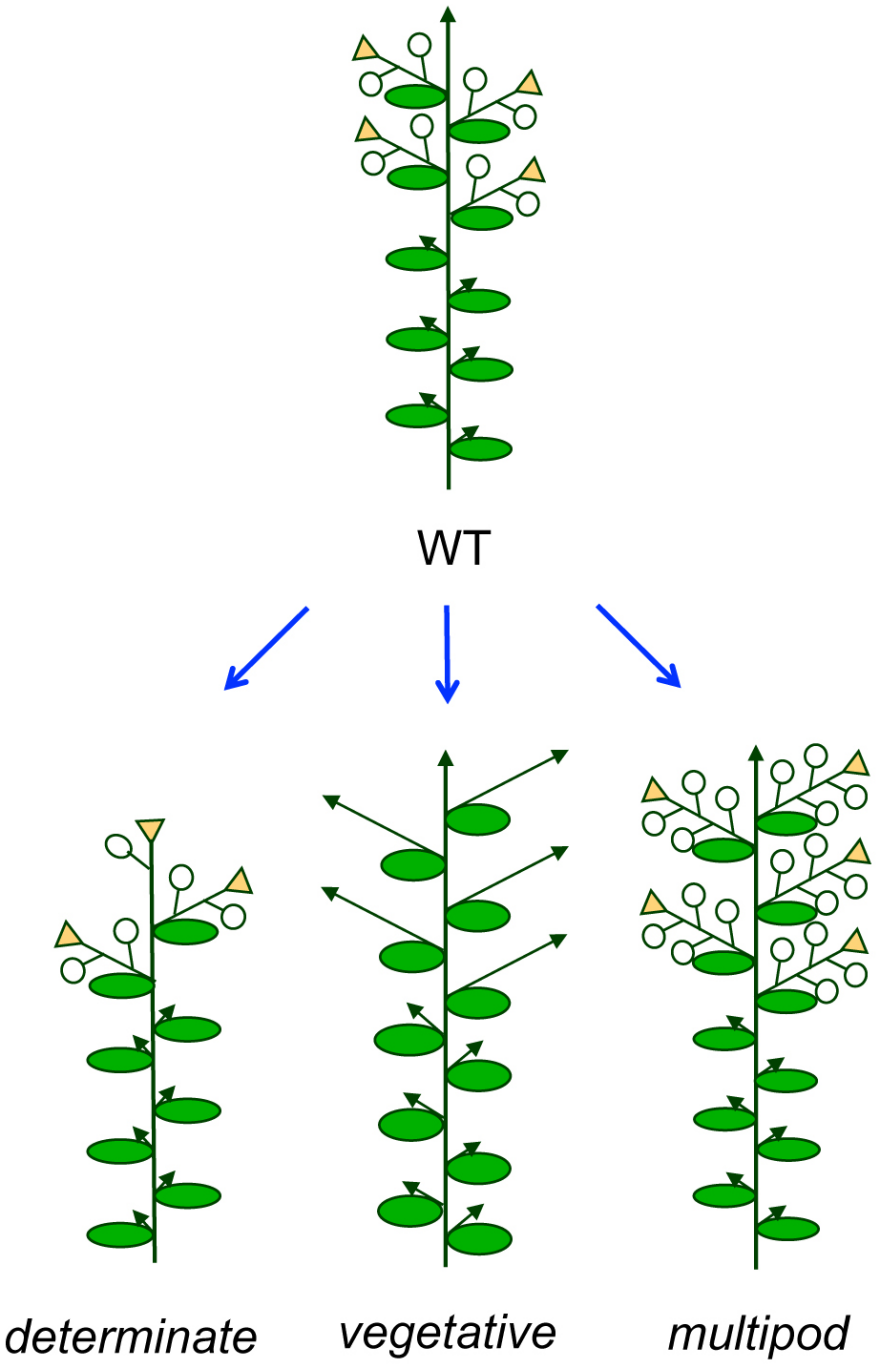
**Possible modifications of the pea inflorescence architecture.** Different plant architectures deriving from modifications of the inflorescence, with potential to improve crop performance in legumes. Open circles represent flowers and arrows represent indeterminate shoots.

At least two main meristem-identity related traits might be amenable to improvement: determinate growth habit and inhibition of flowering (**Figure 4**).

#### Determinate Cultivars

As mentioned before, while wild accessions of most grain legumes have an indeterminate growth habit, where the main (primary) inflorescence meristem continues growing and producing lateral (secondary) inflorescences until its senescence, in many legume crop species determinate varieties exist, in which the growth of the primary inflorescence meristem is interrupted, soon after onset of flowering, by the production of a terminal inflorescence (**Figures 3** and **4**; Singer et al., 1990; Tian et al., 2010; Kwak et al., 2012). Determinate growth habit leads to a reduction in the flowering period, which can be beneficial under certain growing conditions. Thus, under rain-fed conditions the coincidence of growth duration of crop varieties to soil–moisture–availability is essential to reach high seed yields (Siddique et al., 2003) or to avoid lodging in some crops (Duc et al., 2014). Also, determinate cultivars are sometimes preferred because this growth habit facilitates mechanical harvesting (Kelly, 2001; Boote et al., 2003). As described above, recessive mutations in homologs of the *Arabidopsis TFL1* gene underlie this determinate trait, at least in the grain legume crops where the genetic basis of the phenotype has been elucidated (Foucher et al., 2003; Avila et al., 2007; Liu et al., 2010; Repinski et al., 2012; Dhanasekar and Reddy, 2014).

In several legume crops, cultivars with semideterminate growth habit exist, where cessation of growth of the main inflorescence shoot occurs later than in determinate varieties. Advantages of these cultivars are that they produce a larger number of secondary inflorescences (and, therefore, pods) than determinate ones and, at the same time; they are less prone to lodging because they are shorter than indeterminate cultivars (Bernard, 1972; Kapoor and Gupta, 1991). In soybean, semideterminate growth habit has been linked to a dominant mutation leading to high expression in the apex of the main inflorescence of *DT2*, a homolog of the pea I2 meristem identity gene *VEG1* (Ping et al., 2014).

According to what is known, it would seem that isolation of mutants in *TFL1* homologs should be the most direct way to obtain determinate varieties of grain legume crop species. In contrast, it is not clear that a strategy to obtain semideterminate varieties through mutation will be possible because the causative mutation(s) of semideterminacy in soybean has not been fully elucidated yet, and also because they are gain-of-function mutations, most likely in regulatory regions of the *Dt2* gene, which may be difficult to translate to other legume species (Ping et al., 2014). An alternative strategy that might allow obtaining semideterminate varieties in grain legumes would be the generation of plants overexpressing *Dt2/VEG1*.

#### Vegetative Non-Flowering Cultivars

In the case of forage legume crops, such as alfalfa and clovers, breeding is focused in the increase of total biomass (Hayes et al., 2013). An increase of the vegetative portion of the plant can be obtained through a delay in flowering or by inhibition of formation of secondary inflorescences (**Figure 4**). The genetic control of flowering time in legumes is discussed in another review in this number (see Weller and Ortega, 2015, in this Research Topic).

As described above, in the loss-of-function mutants in the *VEG1* gene in pea, formation of secondary inflorescences (I2s) is inhibited, as these structures are transformed into vegetative branches (I1s) (**Figure 3**) and, consistent with that, pea plants where *VEG1* was transiently silenced through virus-induced-silencing (VIGS) produced more vegetative nodes than the WT control (Berbel et al., 2012). As the function of *VEG1* and other factors involved in the same genetic route, as *GIGAS* or *VEG2*, might be conserved in other grain legumes a possible strategy to inhibit flowering in forage legume species could be the isolation of mutants with reduced function of the homologs of these genes (Hecht et al., 2011; Berbel et al., 2012; Sussmilch et al., 2015).

On the other hand, and according to the proposed genetic model, the non-flowering phenotype of pea *veg1* mutants appears to be caused by ectopic expression of the *TFL1*-homolog *DET* gene in all the meristems in the inflorescence apex, which transforms the branches produced in the “inflorescence” apex into primary inflorescences (I1s) and inhibit the formation of flowers (Hecht et al., 2011; Berbel et al., 2012). Therefore, an alternative strategy could be the overexpression of *DET/TFL1* homologs that should reduce *VEG1* activity and might result in inhibition or delay of flowering. This would represent a similar situation to the overexpression of *TFL1* genes in *Arabidopsis* or tobacco, which leads to an extreme inhibition of flowering (Ratcliffe et al., 1998; Amaya et al., 1999).

### Traits Related to the Activity of the Inflorescence Meristems

#### Multiflower/Multipod Pod Cultivars

The number of flowers per I2 (multipod/multiflower) is an inflorescence trait related to the activity of the inflorescence meristem that might be amenable to improvement in grain legumes (**Figure 4**). The possibility of increasing the number of pods appears an attractive option to increase yield in grain legumes. In this sense, in the case of chickpea it has been reported that the double-flower trait has the potential to increase yield (Kumar et al., 2000) or to have a positive effect on yield stability (Rubio et al., 2004). However, translation of this trait to grain legumes different from pea or chickpea is currently limited because the genes responsible of the multiflower/multipod trait have not been identified. Progress on the mapping of the chickpea *SFL* gene, responsible of the double- and triple-flower phenotypes, has been reported, which places *SFL* on LG6, (Rajesh et al., 2002; Gaur et al., 2011). To date, no linkage analysis has been reported for *CYM*, the chickpea gene responsible of the multi-flower phenotype.

Apart from the isolation of mutants for the multiflower/multipod genes, an alternative strategy that also might lead to an increased number of flowers per I2 node could be to manipulate the expression of genes that control general meristem activity. In *Arabidopsis*, a main determinant of shoot and inflorescence meristem activity is the *WUSCHEL* (*WUS*) gene, which codes for a homeobox-type transcription factor that induces stem-cell identity and is essential to maintain the population of stem cells in the meristems. *WUS* expression in the center of the meristems is directly correlated with an active state of stem cells within the meristem, and when *WUS* expression disappears meristems arrest. In *wus* loss-of-function mutants very few flowers are formed and these have a reduced number of floral organs (Mayer et al., 1998). Opposite phenotypes are observed when *WUS* expression is increased as, for instance, in *clavata* mutants that produce more flowers and these flowers have an increased number of floral organs (Schoof et al., 2000).

*WUS* homologs have been described in legumes and the available data suggest that they play the same function in meristem activity as in *Arabidopsis* (Wong et al., 2010). Therefore, it is conceivable that directing the expression of *WUS* to the I2 meristem, for example with the *VEG1* promoter, might lead to an increased activity of the I2 meristem and, therefore to a higher production of flowers.

## Perspectives for Legume Inflorescence Improvement, the Help of Genomics

As shown in the previous sections, we now have a rather good knowledge of the genetic networks that control major traits related to inflorescence architecture in legumes. In some legume species, particularly in pea, key regulators of inflorescence architecture have been isolated and functionally characterized and we can make rather reliable predictions of which of these genes should be used, and how, to improve inflorescence architecture in grain legumes for easier crop management and a higher and stable yield. Nonetheless, much work still needs to be done and genomics and related disciplines will be of great help to speed up progress in this area.

In addition to the sequenced genomes of the model species *L. japonicus* and *M. truncatula* (Sato et al., 2008; Young et al., 2011), genome sequencing has extended in the last years to crop legumes, mainly thanks to the advances in next generation sequencing (NGS) technologies. Thus, genome sequences are now available for soybean, chickpea, and pigeonpea (Schmutz et al., 2010; Varshney et al., 2012, 2013) and those of other important legume crops, such as pea, lentil or peanut are currently underway (Varshney et al., 2014a). The availability of genome sequences and other genomic resources should greatly facilitate the translation of basic knowledge obtained in a few legume models to the breeding of legume crops, and the reader is referred to recently published reviews on this subject for more details (Bolger et al., 2014; Varshney et al., 2014a,b).

Access to the genome sequence of crop legumes will allow easy identification and cloning of the homologs of inflorescence architecture regulators previously identified in model grain legumes such as pea or *Medicago*. Indeed, homologous genes of the major inflorescence regulators can be found in the sequence databases of several grain legumes (**Table 1**), therefore facilitating the identification of suitable alleles to breed inflorescence traits in the legume crop of interest.

**Table 1:** Homologs of main regulators of inflorescence development in legumes.

Gene	Species	Mutant	Gen name/accession (CDS/mRNA)	Reference
*PIM*	*Pisum sativum*	Yes (*pim*)	-/AJ279089	Berbel et al. (2001); Taylor et al. (2002)
	*Medicago truncatula*	Yes (*mtpim*)	Medtr8g066260/DQ139345	Benlloch et al. (2006)
	*Phaseolus vulgaris*	No	Phavu_003G281000/-	
	*Cicer arietinum*	No	-/XM_004509697	
	*Lotus japonicus*	No	-/AY770395 AP1a -/AY770396 AP1b	Dong et al. (2005)
	*Vigna unguiculata*	No	-/AB588744	
	*Glycine max*	No	Glyma16g13070/XM_003547744 Glyma08g36380/XM_003531909 Glyma01g08150/XM_003516406 Glyma02g13420/XM_006574898	Chi et al. (2011)
				
*UNI*	*P. sativum*	Yes (*uni*)	-/AF010190	Hofer et al. (1997)
	*M. truncatula*	Yes (*sgl1*)	-/AY928184	Wang et al. (2008a)
	*P. vulgaris*	No	Phavu_009G160900g/XM_007137786	
	*C. arietinum*	No	-/XM_004501703	
	*L. japonicus*	Yes (*pfm*)	-/AY770393	Dong et al. (2005)
	*M. sativa*	No	-/JF681134	
	*G. max*	No	Glyma06g17170/- Glyma04g37900/- -/DQ448810	
				
*VEG1*	*P. sativum*	Yes (*veg1*)	-/JN974184	Berbel et al. (2012)
	*M. truncatula*	No	Medtr7g16630/XM_003621473	
	*P. vulgaris*	No	Phavu_008G027800g/XM_007139357	
	*C. arietinum*	No	-/XM_004491849 mRNA	
	*L. japonicus*	No	-/BT143167	
	*G. max*	Yes (*dt2*) no	Glyma18g50910/- Glyma08g27680/FG990175	Ping et al. (2014)
				
*GIGAS*	*P. sativum*	Yes (*gigas*)	-/HQ538822	Hecht et al. (2011)
	*M. truncatula*	Yes (*fta1*)	Medtr7g084970/XM_003624521	Laurie et al. (2011)
	*P. vulgaris*	No	Phavu_001G097300g/XM_007161712	
	*C. arietinum*	No	-/XM_004493070	
	*L. japonicus*	*No*	chr1.CM0104.1670.r2m/-	Ono et al. (2010)
	*M. sativa*	No	-/JF681135	
	*G. max*	No	Glyma16g04840/AB550124	Kong et al. (2010)
				
*DET*	*P. sativum*	Yes (det)	-/AY340579	Foucher et al. (2003)
	*M. truncatula*	No	Medtr7g104460/XM_003625760	
	*P. vulgaris*	Yes (fin)	-/JN418230	Repinski et al. (2012)
	*C. arietinum*	No	-/XM_004494111	
	*L. japonicus*	No	-/AY423715	Guo et al. (2006)
	*V. unguiculata*	Yes (TCM418, TCM420, TCM440)	–/KJ569523	Dhanasekar and Reddy (2014)
	*G. max*	Yes (dt1)	Glyma19g37890/AB511820 (GmTFL1b) Glyma03g35250/AB511821 (GmTFL1a)	Liu et al. (2010)
	*Vicia Faba* L.	Yes	-/EF193847	Avila et al. (2007)
	*Cajanus cajan*	No	-/C.cajan_10074	Mir et al. (2014)
				
*LF*	*P. sativum*	Yes (*lf)*	-/AY343326	Foucher et al. (2003)
	*M. truncatula*	No	-/KEH42040	
	*C. arietinum*	No	-/XM_004515550	

Next generation sequencing technologies should also greatly ease the identification of candidate genes for architectural traits whose genetic basis remains unknown. The recently developed mapping-by-sequencing approaches are now starting to be routinely used to rapidly identify candidates for causal mutations in model organisms and, as the NGS technologies become more powerful and cheaper, their use is extending to crop species in spite of their usually larger genomes (Schneeberger et al., 2009; Abe et al., 2012; James et al., 2013). These methods bear great promise for the isolation of novel architecture-related genes identified from forward genetics or natural variation that eventually can be incorporated to breeding strategies.

In addition, different reverse genetic and genomic tools that can be used to validate the function of candidate genes for architectural traits are now available in several model and non-model legume species. First, mutant populations for the retrotransposons *Tnt1*, in *M. truncatula* and *Lore1* in *L. japonicus* are now routinely used for identification of mutants for genes of interest through reverse genetics (Cheng et al., 2011; Urbański et al., 2012). Second, the virus induced gene silencing (VIGS) methods are available to several legume species such as pea, soybean, common bean, *Latirus odorata* and *M. truncatula* (Constantin et al., 2004; Grønlund et al., 2008; Zhang et al., 2010). VIGS is now allowing to successfully analyzing gene function in several legumes, including species that are recalcitrant to genetic transformation (Wang et al., 2008b; Liu et al., 2010). Finally, Targeting Induced Local Lesions In Genomes (TILLING) and EcoTILLING, which allow identification of point mutations and allelic variability in specific genes, can be applied to any species where mutagenised EMS-populations are available or to germplasm collections (Colbert et al., 2001; Comai et al., 2004; Tsai et al., 2011). TILLING platforms are effective reverse genetics tools that already have been proven highly successful for functional analysis of developmental regulators in legumes (Hofer et al., 2009; Berbel et al., 2012). These platforms are currently available for pea, *M. truncatula*, *L. japonicus* and chickpea (Perry et al., 2003; Dalmais et al., 2008; Le Signor et al., 2009; Varshney et al., 2014a), and will likely be developed for other grain legumes as well, providing a rich source of allelic variation for breeding purposes with potential to be used in virtually any diploid crop legume.

Finally, “designer” alleles also appear to be within reach through the recently developed genome editing techniques mediated by the CRISPR-Cas system. CRISPR has been showed to efficiently work in several plants like *Arabidopsis* and rice, where it was possible to engineer site-directed mutations in the genes of interest (Li et al., 2013; Shan et al., 2013; Lozano-Juste and Cutler, 2014). However, while extremely powerful, the use of CRISPR mutagenesis is currently limited to genetically transformable species.

In summary, the increasing availability of genomic tools and resources offers a unique opportunity to accelerate breeding of inflorescence architecture and, in general, of agronomic important traits in legumes. The combination of the increased understanding of the genetic networks controlling legume inflorescence architecture and the use of these genomic tools and resources, promises a rapid progress in obtention of new legume varieties with improved performance, which will be instrumental in developing a sustainable agriculture for the future.

## Conflict of Interest Statement

The authors declare that the research was conducted in the absence of any commercial or financial relationships that could be construed as a potential conflict of interest.
